# Clinical Relevance and Antimicrobial Profiling of Methicillin-Resistant *Staphylococcus aureus* (MRSA) on Routine Antibiotics and Ethanol Extract of Mango Kernel (*Mangifera indica* L.)

**DOI:** 10.1155/2020/4150678

**Published:** 2020-02-18

**Authors:** Ali Al Bshabshe, Martin R. P. Joseph, Amgad A. Awad El-Gied, Abdalla N. Fadul, Harish C. Chandramoorthy, Mohamed E. Hamid

**Affiliations:** ^1^Department of Internal Medicine, College of Medicine, King Khalid University, Abha, Saudi Arabia; ^2^Department of Microbiology and Clinical Parasitology, College of Medicine, King Khalid University, Abha, Saudi Arabia; ^3^Department of Pharmaceutics, College of Pharmacy, King Khalid University, Abha, Saudi Arabia; ^4^Department of Clinical Laboratory Sciences, College of Applied Medical Sciences, King Khalid University, Abha, Saudi Arabia

## Abstract

Methicillin-resistant *Staphylococcus aureus* (MRSA) is known for serious health problems. Testing new inexpensive natural products such as mango kernel (*Mangifera indica* L., Anacardiaceae) may provide alternative and economically viable anti-MRSA drugs. In the current study, we screened clinical isolates from Aseer Central Hospital, Saudi Arabia, during 2012–2017 for MRSA and tested an ethanolic extract of mango kernel for anti-MRSA activity. Brief confirmation of MRSA was performed by the Vitek 2 system, while antibiotic sensitivity of strains was tested for their clinical relevance. The *In vitro* disc diffusion method was used to test the anti-MRSA activity of the ethanolic mango kernel extract. The antimicrobial activity of mango kernel was compared to that of standard drugs (oxacillin and vancomycin). Of the identified 132 *S. aureus* strains, 42 (31.8%) were found to be MRSA and their prevalence showed a clear increase during the last two years (2016-2017; *p* < 0.001). MRSA strains showed 100% sensitivity to vancomycin, teicoplanin, linezolid, tetracycline, daptomycin, tigecycline, and tobramycin and 100% resistance to ampicillin and 98% to penicillin. The ethanolic extracts of mango kernel were found active against both *S. aureus* and the MRSA strains. Inhibitory activities (mean ± SE) were achieved at concentrations of 50 mg/mL (20.77 ± 0.61), 5 mg/mL (16.18 ± 0.34), and 0.5 mg/mL (8.39 ± 0.33) exceeding that of vancomycin (*p*=0.0162). MRSA strains were sensitive to mango kernel extracts when compared to vancomycin. Therefore, ethanolic extracts of mango kernel can be escalated to animal model studies as a promising leading anti-MRSA drug candidate and can be an economic alternative to high-priced synthetic antibiotics.

## 1. Introduction

*Staphylococcus aureus* is a Gram-positive, ubiquitous bacterial pathogen that can perfectly adapt and is capable of living in different states. It has been reported that *S. aureus* can survive in an inanimate environment, existing as a colonizer or commensal, and may form biofilms [1]. *S. aureus* is an important cause of a wide range of clinical infections including bacteraemia and infective endocarditis, osteoarticular, skin and soft tissue, pleuropulmonary, and device-related infections [2]. The spread of methicillin-resistant *S. aureus* (MRSA), especially in nosocomial health settings, is a well-known health threat and economic burden worldwide [3].

The introduction of newer potent systemic antibiotic combinations failed to control the endemic reservoir of multidrug-resistant bacteria and suggests that such policies have little impact [4]. There are reports of increasing drug resistance against human pathogens as well as undesirable side effects of certain antimicrobial agents. The overall global trend shows alarming rise and expansion of MRSA [5]. A meta-analysis showed an obvious connection between exposure to antibiotics and MRSA prevalence, a crucial information which may be considered when planning future MRSA control policies [6]. The literature demonstrates a clinically important burden of *S. aureus* related to various types of infection operations and a substantial contribution of MRSA. Preventive strategies aimed specifically at MRSA could reduce healthcare costs and improve patient outcomes [7, 8]. The prevalence of MRSA is high and rapidly increasing worldwide but is a major public health worry in developing countries. Lack of surveys and collection of epidemiological data and absence of efficient health control programs are contributing to the spread of MRSA [9].

It is necessary to search for new agents that are better, cheaper, and without side effect for treating infectious diseases especially in developing countries. It has been long known that an extensive variety of plant and plant-derived natural products are used in the treatment of infections. Phytoconstituents have been found to inhibit bacteria, fungi, viruses, and pests [10]. The curative effect and appropriateness of products derived from natural plants against diseases are a recognized and documented reality. *Mangifera indica* (mango kernel) is an example which was recorded to have antimicrobial activities. Application of such innovative results may help in controlling the infection and preventing their spread.

Previous studies have shown that extracts of mango kernel are promising candidates against numerous species of pathogenic bacteria [11–13]. Leaves of mango kernel have been reported to possess antibacterial activity against *Escherichia coli* including members of *Enterobacteriaceae* family, while mangiferin, a bioactive component, has been reported to possess remarkable anti-influenza activity [14]. Both acetone and methanol extracts inhibited the growth of Gram-positive bacteria, with a zone of inhibition between 15 and 16 mm and a zone of 14 mm to Gram-negative bacterium *Salmonella typhi* at 250 mg/mL. Thai mango (*M. indica* L. cv. “Fahlun”) seed kernel extract was found to have potent antimicrobial properties [15]. Aqueous and chloroform extracts of the leaves and stem of mango kernel were found to be active as an antibacterial agent against *S. Aureus* [16]. Mangiferin was found to have antibacterial activity in vivo against specific periodontal pathogens such as *Actinobacillus actinomycetemcomitans, Prevotella intermedia, Porphyromonas gingivalis, Fusobacterium nucleatum*, and *Peptostreptococcus* sp. It has been suggested to be used as an alternative remedial agent or an adjunctive treatment along with classic antibiotics [15, 17].

This study aimed to examine the clinical significance of MRSA and to determine their antimicrobial profile against routine antimicrobial agents and against ethanolic extracts of mango kernel.

## 2. Materials and Methods

### 2.1. Study Design

This study was conducted between 2012 and 2017 at definite time intervals and duration at the Department of Microbiology and Clinical Parasitology, College of Medicine, King Khalid University, and Aseer Central Hospital, Abha, Saudi Arabia. Ethical approval was obtained from the Ethics Committee of the College of Medicine, King Khalid University, vide reference letter, REC # 2017-02-17. Patient information, clinical data, and bacteriological and antimicrobial records of the isolates were collected and saved in a spreadsheet for analysis.

### 2.2. Inclusion and Exclusion Criteria

All isolates were Gram-positive coccus and catalase- and coagulase-positive. Coagulase-negative staphylococci and duplicate isolates were excluded. Both MRSA and other coagulase-positive *S. aureus* strains were included in the study for comparative reasons.

### 2.3. Bacterial Isolates

*S. aureus* strains (*n* = 132) were isolated from various clinical samples of patients attending Aseer Central Hospital, Abha, Saudi Arabia. Samples were processed for culture by standard conventional methods, and initial identification was performed by standard phenotypic tests [18, 19]. Isolates that were Gram-positive coccus and catalase- and coagulase-positive were collected. Subsequent initial bench identification confirmation of MRSA was done at the microbiology laboratory using the Vitek 2 identification system (BioMerieux, France) according to the manufacture standards.

### 2.4. Antibiotic Susceptibility Testing (AST)

Antimicrobial susceptibility testing was carried out using the Vitek 2 system, according to the manufacturer's information. The following antimicrobial agents have been tested for at least one or more strains: Amikacin; Amox/K Clav; Amp/Subactm; Ampicillin; Azithromycin; Cefepime; Cefotaxime; Cefoxitin; Cefuroxime; Cephalothin; Chloramphenicol; Ciprofloxacin; Clarithromycin; Clindamycin; Colistin; Cotrimox; Daptomycin; Erythromycin; Fosfomycin; Fusidc Acid; Gentamicin; Imipenem; Levofloxacin; Linezolid; Meropenem; Moxifloxacin; Mupirocin; Nalidixic; Neomycin; Nitrofurantoin; Norfloxacin; Ofloxacin; Oxacill/Methicill; Oxacillin; Penicillin; Pip/Tazo; Piperacillin; Rifampin; Sulfamethoxazole; Synercid; Teicoplanin; Tetracyclin; Tigecycline; Tobramycin; Trimeth/Sulfa; and Vancomycin.

### 2.5. Oxacillin Screen

Oxacillin screen agar testing was performed for *S. aureus* according to the NCCLS methodology [20] using Mueller–Hinton agar with 4% NaCl and oxacillin (6 *μ*g/ml). Plates were inoculated with the 0.5 McFarland bacterial suspensions and incubated at 35°C for 24 h.

### 2.6. Plant Collection and Identification

Fresh mango kernel was collected from local markets in Abha, Saudi Arabia and was identified and authenticated at the Department of Biological Sciences, College of Science, King Khalid University. The collected seeds were stored in polythene bags at room temperature till further use.

### 2.7. Extraction of Plant Seeds

Mango kernel seeds were cleaned, air-dried, and coarsely powdered. 1000 grams of powder was extracted with ethanol in a Soxhlet apparatus. The extracts were filtered, and the filtrates were vaporized to dryness and weighed in order to determine the % yield of the extracts, following the formula: % yield = (weight of extract/weight of ground plant material) × 100. The stock solutions of the crude ethanolic extracts were prepared by dilution of the dried extracts with 50% ethanol to obtain the desired final concentrations of 50 mg/mL, 5 mg/mL, 0.5 mg/mL, and 0.05 mg/mL. These concentrations were used to impregnate filter paper disks (5.5 mm diameters).

Disks impregnated into 50% ethanol were used as control, while standard antimicrobial disks such as oxacillin and vancomycin (Difco) were used as positive control.

### 2.8. Antimicrobial Assays of Plant Extracts against MRSA

MRSA bacterial strains (*n* = 51) recovered from clinical samples isolated from patients at Aseer Central Hospital were used in the study. One to three loopful of 24 h old cultures from each test strains were used to prepare 0.5 McFarland standard suspensions.

### 2.9. Determination of MIC and MBC Values

Minimum inhibitory concentration (MIC) of ethanolic extraction of mango kernel was determined by using descending concentrations of the extract. The MIC of the extract was diluted using sterile saline and was tested for antibacterial activity against *Staphylococcus aureus* and MRSA according to [21] with some modifications. The different prepared concentrations were tested against the bacterial strains using disc diffusion assay as previously mentioned. The formed clear zones were measured and recorded. Serial dilution was determined starting with a concentration of 1 mg/mL in the tubes containing 2 mL of sterile Mueller–Hinton broth and discarded 2 mL from the final tube. One tube with Mueller–Hinton broth as positive control and one tube with Mueller–Hinton broth as negative control were used for the test. 0.2 mL of bacterial suspension ranging from 0.5 to 1 Mcfarland was added to all tubes except to the negative control tube, and all the tubes were incubated overnight at 37°C aerobically. Duplication of each MIC test was performed. After the overnight incubation, subculture of each tubes was prepared by the conventional plating method on Mueller–Hinton agar to examine the growth or no growth, and MIC of the test was calculated as the mean of lowest concentration of inhibition and the highest concentration allows the growth of the test microorganism.

### 2.10. Statistical Analysis

Statistical analysis of data obtained from triplicate experiments was performed using SPSS software version 16.0. The nonparametric test for several independent samples was used to compare variation among means of different inhibition zones induced by extracts in comparison with positive controls. Linear regression was applied to assess the relationships among several independent samples. *p* value <0.05 was considered as the level of significance.

## 3. Results

### 3.1. Prevalence of MRSA and *S. aureus* Strains

**Figure 1:**
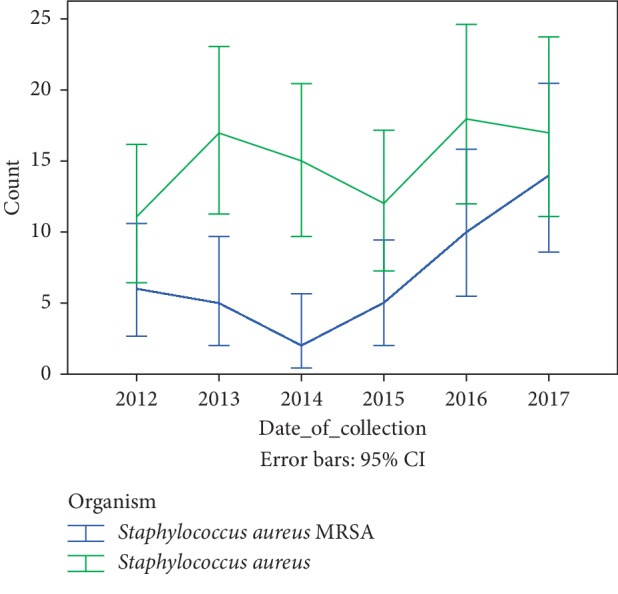
Prevalence of MRSA and other *S. aureus* strains isolated from various clinical samples of patients attending Aseer Central Hospital, Abha, Saudi Arabia in five years (2016-2017).

Prevalence of MRSA and other *S. aureus* strains isolated from various clinical materials of patients attending Aseer Central Hospital, Abha, Saudi Arabia in five years (2016-2017) is shown in Figure 1. Of the 132 suspected *S. aureus* strains, 42 (31.8%) were found to be MRSA and the rest (68.2%) were *S. aureus*. The prevalence of MRSA demonstrated an obvious rise during 2016-2017 compared to the previous three years (*p* < 0.001).

### 3.2. Distribution of MRSA and *S. aureus* Strains according to Clinical Specimens and Age Groups

MRSA and other *S. aureus* strains originated from all age groups, but the majority was from 20- to 40-year-old patients. The strains were recovered predominantly from the respiratory specimens (77.3%) including endotracheal tube (53.8%), throat swab (10.6%), sputum (6.8%), and tracheal secretion (5.3%), followed by blood (10.6%) and wound specimens (4.5%).

### 3.3. Susceptibility of MRSA and *S. aureus* Strains to Routine Antimicrobials

**Figure 2:**
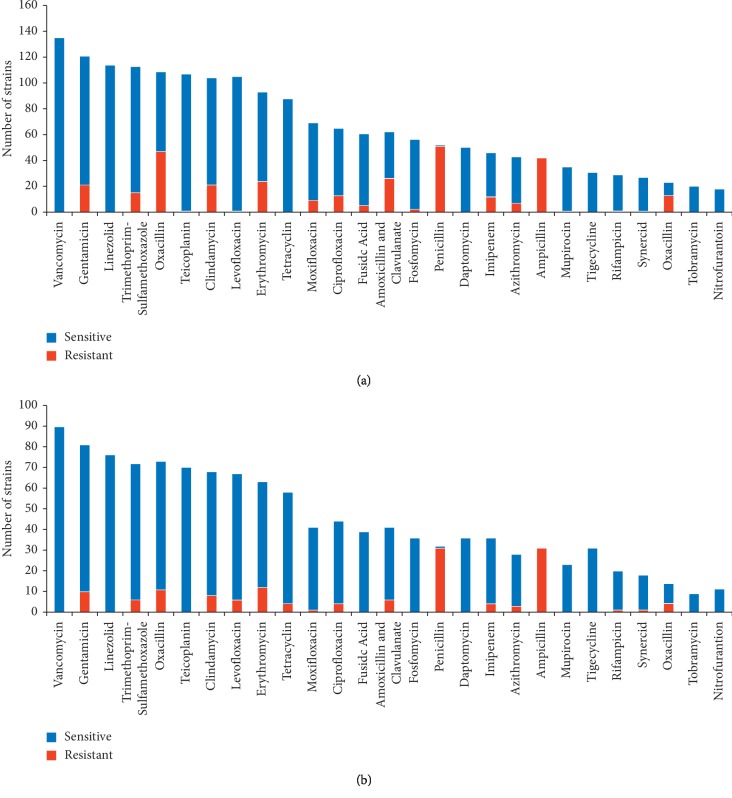
Susceptibility of MRSA and *S. aureus* strains to routine antimicrobials. Strains were isolated from various clinical samples of patients attending Aseer Central Hospital, Abha, Saudi Arabia, in five years (2016-2017). (a) Methicillin-resistant *Staphylococcus aureus* (MRSA). (b) Methicillin-sensitive *Staphylococcus aureus* (MRSA).

The susceptibility of MRSA and *S. aureus* strains to routine antimicrobials is shown in Figure 2. MRSA strains showed 100% sensitivity to vancomycin, teicopanin, linezolid, tetracycline, daptomycin, tigecycline, and tobramycin and more than 99% sensitivity to teicoplanin and levofloxacin. On the other hand, MRSA strains showed 100% resistance to ampicillin and 98% resistance to penicillin. The ethanolic extracts of mango kernel were found to be active against both *S. aureus* and the MRSA strains. Inhibitory activities (mean ± SE) were achieved at concentrations of 50 mg/mL (20.77 ± 0.61), 5 mg/mL (16.18 ± 0.34), and 0.5 mg/mL (8.39 ± 0.33) but not at 0.05 mg/mL (0 ± 0.00). Inhibition zone by oxacillin was 9.45 mm while vancomycin was 20 ± 0.24 mm. The MIC was found (0.15625 mg/mL), whereas the MBC was (0.25 mg/mL). The antagonistic value of mango kernel at 50 mg/mL exceeded that of vancomycin (*p*=0.0162) but at 5 mg/mL was found to be less active than vancomycin (*p* < 0.001); nevertheless, at this concentration it exceeded oxacillin (*p* < 0.001).

### 3.4. Susceptibility of MRSA and *S. aureus* Strains to Various Concentrations of Ethanolic Extract of Mango Kernel

The susceptibility of MRSA and *S. aureus* strains to various concentrations of ethanolic extract of mango kernel is shown in Table 1.

**Figure 3:**
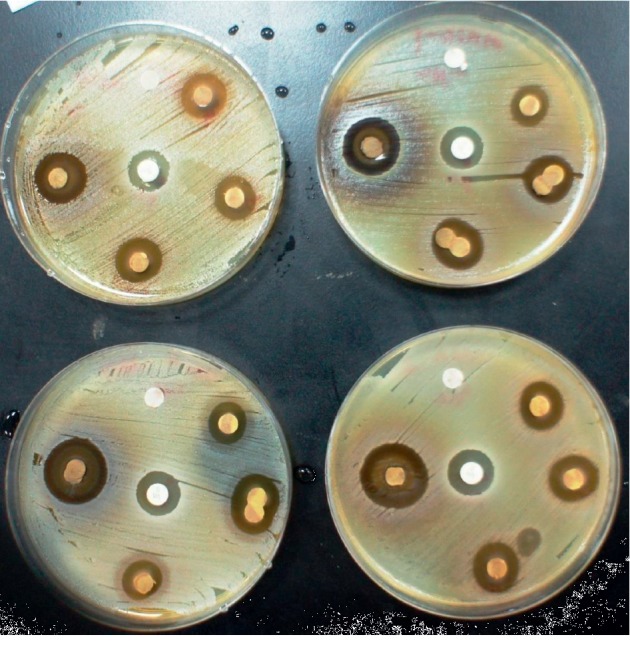
Inhibitory activities (clearance) of ethanolic extracts of mango kernel against both *S. aureus* and the MRSA strains.

The ethanolic extracts of mango kernel were found active against both *S. aureus* and the MRSA strains (Figure 3). Inhibitory activities (mean ± SE) were achieved at concentrations 50 mg/mL (20.77 ± 0.61), 5 mg/mL (16.18 ± 0.34), and 0.5 mg/mL (8.39 ± 0.33) but not at 0.05 mg/mL (0 ± 0.00). The inhibition zone by oxacillin was 9.45 mm while the inhibition zone by vancomycin was 20 ± 0.24 mm. The MIC was found to be 0.15625 mg/mL, whereas the MBC was 0.25 mg/mL.

**Figure 4:**
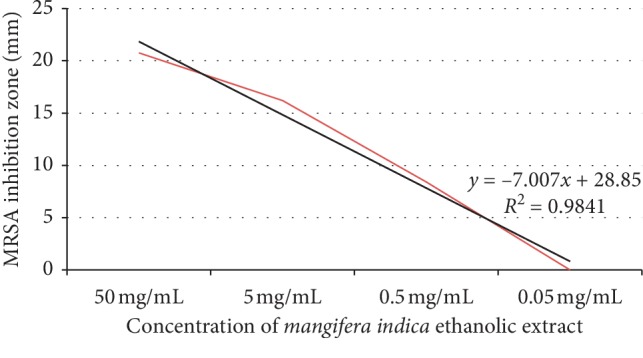
Linear regression curve illustrating activity of various concentrations of ethanolic extract of mango kernel against *S. aureus* and MRSA.

A linear regression curve illustrating the activity of various concentrations of ethanolic extract of mango kernel against *S. aureus* and MRSA is shown in Figure 4.

**Figure 5:**
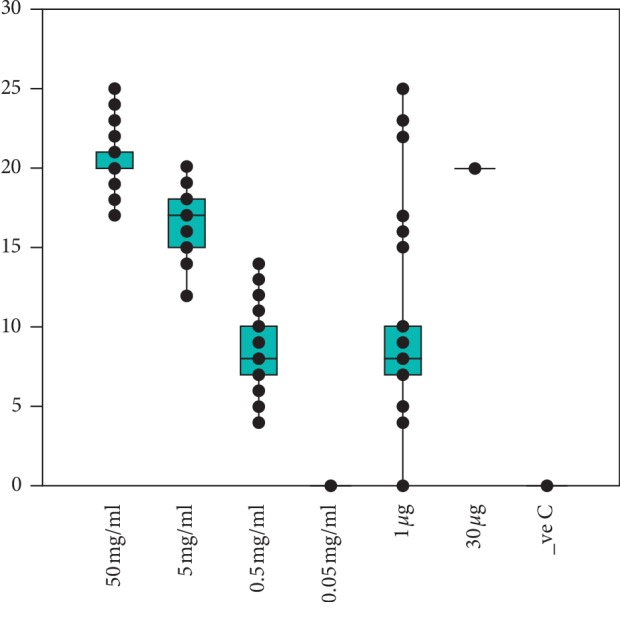
Antagonistic value of mango kernel at various concentrations compared to that of vancomycin and oxacillin.

The antagonistic value of mango kernel at 50 mg/mL exceeded that of vancomycin (*p*=0.0162) but at 5 mg/mL, it was found to be less active than vancomycin (*p* < 0.001); nevertheless, at this concentration, it exceeded oxacillin (*p* < 0.001) (Table 2; Figure 5).

## 4. Discussion

The prevalence of MRSA is high and rapidly increasing worldwide but is a major public health worry in developing countries. MRSA is one of the important pathogens that result in heavy health and economic burden throughout the world [8]. But the actual effect of multidrug-resistant bacterial infections on real-world healthcare resources is not fully determined [22]. The spread of MRSA is becoming a well-known phenomenon and rapidly spreading worldwide. Roughly, the cost of MRSA treatment is about US8000 per case and varied in other developing countries [23].

Lack of surveys and collection of epidemiological data and absence of efficient health control programs are contributing to the spread of MRSA [9, 24]. Though our report of MRSA is a little bit on higher side, the average data of Saudi Arabia are as comparable with the global data [25]. The results of the current study showed that testing and application innovative “safe” products like black seed or mango seeds, which have been recently recognized to have antimicrobial activities, can be an economic alternative to control these menacing microbes. Further this will prevent of the spread of MRSA in the hospitals and patients undergoing treatment as a priority to avoid serious consequences.

MRSA is a major contributor and huge burden in the management of nosocomial disease. The overall global trend displays a disturbing rise of MRSA infections in addition to increased occurrence of multidrug resistance (MDR) strains [5]. We had predominantly isolates from respiratory specimens which were well observed in many studies. Our observation of the about 10% from blood well conceded with studies which showed the MRSA was only next to the third-generation cephalosporin-susceptible Enterobacteriaceae. However MRSA had greater impact on mortality, excess length of stay and cost more than Enterobacteriaceae infection [8]. Owing to high incidence of antibiotic-resistant microbes, natural products and herbal extracts previously reported for bioactivity have been seen as inexpensive and low or no side effects lead drugs [26]. They are therefore can be considered appropriate and economic alternative for healing drug resistant infectious diseases [27].

The antibiotic sensitivity pattern of the all the MRSA isolates were comparable thought the study period while antibiotic-resistant bacteria, including community-acquired and hospital-acquired MRSA, vancomycin-intermediate *S. aureus*, vancomycin-resistant enterococci, macrolide- and penicillin-resistant *Streptococcus pneumoniae*, extend-spectrum beta-lactamase-producing *Escherichia coli* and *Klebsiella pneumoniae*, carbapenem-resistant *Enterobacteriaceae*, and multidrug-resistant *Pseudomonas aeruginosa* and *Acinetobacter* spp., are becoming prevalent in many Asian countries including Saudi Arabia [24]. Our earlier study revealed that *Escherichia coli* and staphylococci were the main etiological agents of infections in Aseer region, while purulent exudates of wounds and abscesses were the main source of *S. aureus*. Also, a higher rate of MRSA was detected [25].

The antimicrobial resistance among these pathogens in Saudi Arabia in the last decades and their antimicrobial resistance has increased among *S. aureus* in the Kingdom with a growing prevalence of both nosocomial and community MRSA isolates [28]. The anti-MRSA results over the clinical isolates of MRSA were promising with high discriminatory zone of inhibition between control antibiotics and extracts. The results were comparable with other similar studies done with mango kernel extracts [29]. Further based on the sensitivity pattern no vancomycin-resistant MRSA have been reported, although isolates with reduced susceptibility to the drug was noted. MRSA from KSA were found linked to epidemic strains from the Middle East and possibly India, rather than to the Western European UK countries [30]. High-resolution typing methods, including SCCmec subtyping, might help to differentiate related epidemic strains and to monitor routes of transmission to identify the origin of antibiotic resistance and rational use new drugs.

## 5. Conclusion

Ethanolic extracts of mango kernel showed a significant activity against MRSA isolates. We did not observe any variation in anti-MRSA activity over five years of time nor varied isolates. Mango kernel extracts could be the best and economic alternative to combat growing antibiotic resistance observed thoughout the Middle East and Saudi Arabia. The study could be escalated to small animal models addressing the *in vivo* actions including toxicity, dose, and mechanism of anti-MRSA activity of mango kernel extracts.

## Figures and Tables

**Table 1:** Susceptibility of MRSA and *S. aureus* to various concentrations of ethanolic extract of mango kernel.

SN	Strain	Source	Inhibition (mm)						
			*M. indica*				Oxacillin (mm)	Vancomycin (mm)	
			50 mg/mL	5 mg/mL	0.5 mg/mL	0.05 mg/mL	1 *μ*g	30 *μ*g	Negative control (ethanol-impregnated discs)
1	*S. aureus*	ATCC 25923^*∗*^	21	14	9	0	23	20	0
2	MRSA	Strain 367^*∗∗*^	25	15	7	0	7	19	0
3	MRSA	Strain 1652	20	14	6	0	9	21	0
4	MRSA	Strain R68	24	17	7	0	8	22	0
5	MRSA	Strain 1205	24	18	9	0	8	18	0
6	MRSA	Strain 300	24	19	10	0	8	20	0
7	MRSA	Strain 301	24	18	10	0	9	19	0
8	MRSA	Strain 475	23	17	10	0	8	21	0
9	MRSA	Strain 2	20	18	8	0	8	22	0
10	MRSA	Strain CL2	20	17	8	0	9	18	0
11	MRSA	Strain 396	19	18	8	0	10	20	0
12	MRSA	Strain 682	20	18	9	0	7	19	0
13	MRSA	Strain 6821	23	19	8	0	5	21	0
14	*S. aureus*	Strain 1946	19	17	13	0	17	22	0
15	MRSA	Strain 1947	20	16	11	0	16	18	0
16	MRSA	Strain 1533	25	20	13	0	0	20	0
17	MRSA	Strain 634	20	14	12	0	8	19	0
18	*S. aureus*	Strain 3966	20	14	12	0	16	21	0
19	MRSA	Strain 491	19	17	10	0	8	22	0
20	MRSA	Strain S77	20	12	8	0	5	18	0
21	*S. aureus*	Strain 528	23	18	12	0	25	20	0
22	MRSA	Strain CL1	20	17	6	0	10	19	0
23	MRSA	Strain E2	20	18	14	0	9	21	0
24	MRSA	Strain 19657	21	19	8	0	4	22	0
25	MRSA	Strain 688	17	15	7	0	10	18	0
26	MRSA	Strain 677	20	17	8	0	10	20	0
27	MRSA	Strain 475	23	19	8	0	8	19	0
28	*S. aureus*	Strain 1890	19	17	8	0	15	21	0
29	*S. aureus*	Strain 3889	18	14	5	0	22	22	0
30	MRSA	Strain 9728	20	15	5	0	8	18	0
31	MRSA	Strain 715	20	17	9	0	7	20	0
32	MRSA	Strain 369	20	18	10	0	7	19	0
33	MRSA	Strain 319	21	12	4	0	5	21	0
34	MRSA	Strain S-66	20	17	7	0	8	22	0
35	MRSA	Strain S-85	21	19	7	0	7	18	0
36	*S. aureus*	Strain 1562	20	17	5	0	15	20	0
37	*S. aureus*	Strain 109	20	15	8	0	17	19	0
38	MRSA	Strain 394	20	16	10	0	7	21	0
39	MRSA	Strain 3941	20	17	11	0	8	22	0
40	MRSA	Strain 3677	22	15	7	0	7	18	0
41	MRSA	Strain 3678	21	15	8	0	7	20	0
42	MRSA	Strain 3679	20	14	7	0	8	19	0
43	MRSA	Strain 3680	20	15	7	0	8	21	0
44	MRSA	Strain 3689	21	15	8	0	7	22	0
45	MRSA	Strain 3687	20	14	7	0	8	18	0
46	MRSA	Strain 3686	20	15	7	0	8	20	0
47	MRSA	Strain 3685	21	15	8	0	7	19	0
48	MRSA	Strain 3681	20	14	7	0	8	21	0
49	MRSA	Strain 3682	20	15	7	0	8	22	0
50	MRSA	Strain 3684	21	15	8	0	7	18	0
51	MRSA	Strain 3688	20	14	7	0	8	20	0

Abbreviations: *S. aureus*, *Staphylococcus aureus*; MRSA, methicillin-resistant *Staphylococcus aureus*. ^*∗*^ATCC, American Type Culture Collection; ^*∗∗*^strains originated from clinical materials, Aseer Central Hospital, Abha, Saudi Arabia.

**Table 2:** Summary of descriptive statistics of different concentrations of ethanolic extracts of mango kernel were found to be active against both *S. aureus* and the MRSA strains.

Parameters	50 mg/mL	5 mg/mL	0.5 mg/mL	0.05 mg/mL	1 *μ*g	30 *μ*g
Number of strains	51	51	51	51	51	51
Minimum inhibition zone	17.00	12.00	4.00	0.00	0.00	18.00
Maximum inhibition zone	25.00	20.00	14.00	0.00	25.00	22.00
Mean inhibition zone	20.76	16.18	8.39	0.00	9.45	20.00
Standard error	0.24	0.27	0.30	0.00	0.67	0.20
Standard deviation	1.73	1.92	2.17	0.00	4.75	1.41

## Data Availability

The data have been incorporated in the manuscript. If further information is required, the authors will furnish the additional supporting data.

## Additional Points

*Highlights*. The majority of MRSA strains were from the respiratory system, sensitive to vancomycin but resistant to ampicillin and penicillin. All MRSA strains were sensitive to mango kernel extract and showed *in vitro* susceptibility which matched that of vancomycin. This is a promising therapeutic product and can be used as an economic adjunct treatment immediately.
